# Supplementary material for: Heterologous ChAdOx1 nCoV-19/BNT162b2 Prime-Boost Vaccination Induces Strong Humoral Responses among Health Care Workers
Source: Vaccines (Basel). 2021 Aug 4;9(8):857. doi: 10.3390/vaccines9080857 (PMC8402499; doi:10.3390/vaccines9080857)
Supplement: Supplementary file 1 [file vaccines-09-00857-s001.zip › vaccines-1289519-supplementary.pdf]

vaccines

Supplemental Material

Table S1. Cutoff values of the SARS-CoV-2 specific bead-based multiplex assay.

Target	Cutoff (MFI)
SARS-CoV-2 Full Spike	6800
SARS-CoV-2 Spike S1	2700
SARS-CoV-2 Spike RBD	3800
SARS-CoV-2 Spike S2	3200
SARS-CoV-2 Nucleocapsid Protein	5900
HCoV-229E Spike S1	8012
HCoV-HKU1 Spike S1	4235
HCoV-NL63 Spike S1	4407
HCoV-OC43 Spike S1	3599
MERS-CoV Spike S1	21
SARS-CoV-1 Spike S1	41

SARS, severe acute respiratory syndrome; MERS, middle east acute respiratory syndrome; MFI, mean fluorescence intensity

Table S2. Participants Baseline Characteristics for analysis against different SARS-CoV-2 target antigens.

	AZ/BNT Heterol Boost	BNT/BNT Homol Boost	*P*-value
N	15	15	
Age, median (IQR)	33 (27 – 39)	42 (30 – 56)	0.07
Female, N (%)	7 (47)	7 (47)	>0.99

AZ=AstraZeneca; BNT=BioNTech, N=number

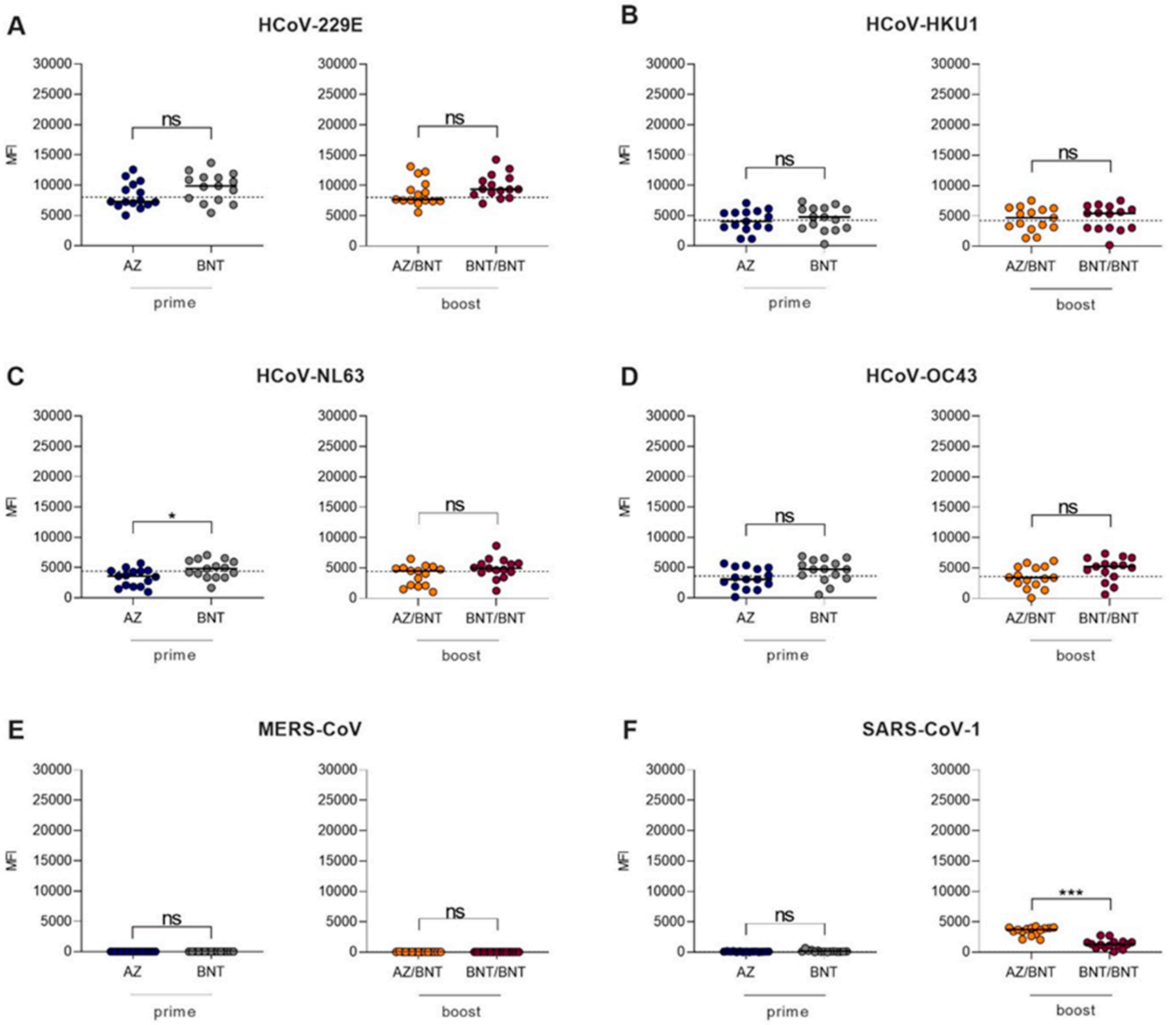

Figure S1. Antibodies against the S1 spike protein of the 4 community coronaviruses, MERS-CoV, and SARS-CoV-1 after AZ and BNT prime vaccination and after AZ/BNT heterologous and BNT/BNT homologous boost vaccination. IgG antibodies against the 4 community coronaviruses, MERS-CoV, and SARS-CoV-1 in health care workers after AZ and BNT prime vaccination and after AZ/BNT heterologous and BNT/BNT homologous boost vaccination. Detection of antibodies against HCoV-229E (A), HCoV-HKU1 (B), HCoV-NL63 (C), HCoV-OC43 (D), MERS-CoV (E), and SARS-CoV-1 (F) in health care workers after AZ and BNT prime vaccination and after AZ/BNT heterologous and BNT/BNT homologous boost vaccination. The x-axis represents the vaccination pattern, and the y-axis the mean fluorescence intensity (MFI) value of the reactivity. The dashed black line represents the cutoff for detection for each target, respectively.

vaccines

Supplemental Methods

Calculation of inhibition via optical density

Optical density at 450 nm was measured in each well and the percent (%) inhibition was calculated as follows:

$$\textbf{Inhibition} = \left(1 - \left(\frac{\textbf{OD value of Sample}}{\textbf{OD value of Negative Control}}\right)\right) \textbf{X 100\%}$$

Side effects questionnaire

1. **Side effects after 1st vaccination:**

◯ yes ◯ no

2. **If yes, please mark the appropriate side effects you had:**

◯ local events (such as pain at injection site, redness, swelling)
◯ fever ◯ fatigue ◯ headache ◯ chills
◯ vomiting ◯ diarrhea ◯ muscle pain ◯ joint pain
◯ swollen lymph nodes ◯ others, such as ___________________

3. **In response to above mentioned side-effects, I took the following medication**

◯ NSAID (i.e. Ibuprofen)
◯ Paracetamol
◯ Metamizol (i.e. Novalgin)
◯ Aspirin
◯ others, such as ___________________

1. **Side effects after 2nd vaccination:**

◯ yes ◯ no

2. **If yes, please mark the appropriate side effects you had:**

◯ local events (such as pain at injection site, redness, swelling)
◯ fever ◯ fatigue ◯ headache ◯ chills
◯ vomiting ◯ diarrhea ◯ muscle pain ◯ joint pain
◯ swollen lymph nodes ◯ others, such as ___________________

3. **In response to above mentioned side-effects, I took the following medication**

◯ NSAID (i.e. Ibuprofen)
◯ Paracetamol
◯ Metamizol (i.e. Novalgin)
◯ Aspirin
◯ others, such as ___________________
